# Electronic and other new media technology interventions for HIV care and prevention: a systematic review

**DOI:** 10.1002/jia2.25439

**Published:** 2020-01-07

**Authors:** Kevin M Maloney, Anna Bratcher, Ryan Wilkerson, Patrick S Sullivan

**Affiliations:** ^1^ Department of Epidemiology Emory University Atlanta GA USA; ^2^ Department of Epidemiology University of California Los Angeles CA USA

**Keywords:** eHealth, mobile applications, smartphones, social media, primary prevention, secondary prevention

## Abstract

**Introduction:**

Electronic and other new media technologies (eHealth) can facilitate large‐scale dissemination of information and effective delivery of interventions for HIV care and prevention. There is a need to both monitor a rapidly changing pipeline of technology‐based care and prevention methods and to assess whether the interventions are appropriately diversified. We systematically review and critically appraise the research pipeline of eHealth interventions for HIV care and prevention, including published studies and other funded projects.

**Methods:**

Two peer‐reviewed literature databases were searched for studies describing the development, trial testing or implementation of new technology interventions, published from September 2014 to September 2018. The National Institutes of Health database of grants was searched for interventions still in development. Interventions were included if eHealth was utilized and an outcome directly related to HIV treatment or prevention was targeted. We summarized each intervention including the stage of development, eHealth mode of delivery, target population and stage of the HIV care and prevention continua targeted.

**Results and discussion:**

Of 2178 articles in the published literature, 113 were included with 84 unique interventions described. The interventions utilize a variety of eHealth technologies and target various points on the prevention and care continua, with greater emphasis on education, behaviour change and testing than linkage to medical care. There were a variety of interventions for HIV care support but none for PrEP care. Most interventions were developed for populations in high income countries. An additional 62 interventions with funding were found in the development pipeline, with greater emphasis on managing HIV and PrEP care.

**Conclusions:**

Our systematic review found a robust collection of eHealth interventions in the published literature as well as unpublished interventions still in development. In the published literature, there is an imbalance of interventions favouring education and behaviour change over linkage to care, retention in care, and adherence, especially for PrEP. The next generation of interventions already in the pipeline might address these neglected areas of care and prevention, but the development process is slow. Researchers need new methods for more efficient and expedited intervention development so that current and future needs are addressed.

## 1 Introduction

Care and prevention interventions for HIV stand at the intersection of two important trends. First, the models of care for people living with HIV and those at risk for HIV have become increasingly reliant on biomedical interventions (HIV treatment and pre‐exposure prophylaxis (PrEP)), and have converged on a conceptual framework of care continua (the HIV care continuum and the PrEP continuum) 1, 2, 3, 4. The care continua are tools to measure the proportion of the population aware of their HIV status and engaged in various stages of HIV or PrEP care, including linkage to care, initiation of treatment, retention in care, adherence, and suppression (for people living with HIV). Second, there has been a proliferation of technology‐based delivery mechanisms for treatment and prevention, including electronic (eHealth) and mobile (mHealth) technology approaches. Mobile and other emerging electronic technologies, collectively referred to hereafter as eHealth, are attractive tools for health communication and innovation of traditional HIV interventions. These technologies can facilitate large‐scale dissemination of information and effective delivery of tools to promote and maintain behavioural modification, routine testing of HIV among uninfected individuals, and linkage to HIV treatment or PrEP. Examples include smartphone applications (apps) for locating HIV testing services, web‐based modules to improve communication in sexual partnerships, and digital games to educate adolescents about substance use and HIV risk. A recent review of eHealth interventions related to HIV prevention and care through 2014 catalogued publications about interventions in development, and noted trends towards use of social networking sites, real‐time assessment and feedback, and gamification 5.

**Figure 1:**
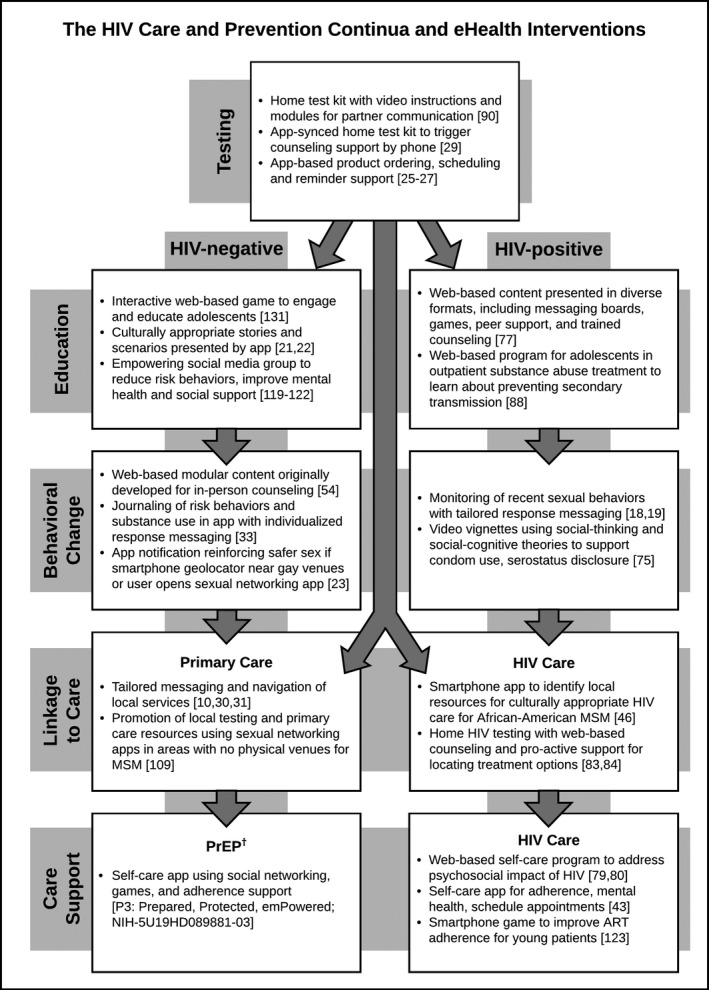
Target points on the HIV care and prevention continua with examples of interventions. Examples of eHealth interventions found in a systematic review of peer reviewed literature (2014–2018). †Denotes an unpublished intervention in the research pipeline.

At the crossroads of a maturing conception of serostatus‐neutral HIV care and prevention and rapidly changing technology, there is a need to both monitor a rapidly changing pipeline of technology‐based prevention methods, and to assess whether the research being conducted is appropriately distributed across the care and prevention continua. For example care and prevention indicators typically monitor distal phases of the continua (e.g. HIV viral suppression for people living with HIV; PrEP uptake for HIV‐negative people at risk for HIV) 6, 7. In the United States, only 51% of people living with HIV aged ≥13 years are virally suppressed 8, 9, and <5% of men who have sex with men (MSM) with PrEP indications have used PrEP 10. But interventions might need to address more proximal aspects of the continua (e.g. linkage to care, increasing awareness of PrEP), and it is important to examine the adequacy of the pipeline of interventions addressing these proximal steps in the continua. To illustrate this, we present HIV care and prevention continua, combining elements of the HIV care and PrEP continua with traditional approaches to HIV prevention, including testing, education and behavioural change. We have provided an illustration of these continua and examples of interventions (Figure 1).

We conducted a systematic review of published literature and of public data on funded NIH grants to describe technology‐based interventions for HIV care and prevention, and to assess the relative distribution of those interventions on the care and prevention continua.

## 2 Methods

We conducted a systematic review of published literature and publicly funded research, updating the methodology outlined by Muessig et al to assess what progress has been made since their systematic review of the literature within the framework of the prevention and care continua 5. Briefly, we searched the PubMed and Web of Science databases using the dates 1 September 2014 to 1 September 2018 with the search term “HIV” in combination with any of the following: “mobile health”; “mHealth”; “mobile phone”; “cell phone”; “smartphone”; “social media”; “mobile application”; “app”; “eHealth”; “Internet”; or “game”. Article titles and abstracts were reviewed for relevance, and candidate articles were reviewed in full for final inclusion in the study.

We selected papers for which an intervention was clearly described, piloted or tested, including research and development protocols, with a primary or secondary outcome directly related to HIV treatment or prevention. We defined “intervention” broadly, to include any eHealth resource, tool or product which can be used to educate, motivate behaviour change, link users to external resources and/or support prevention activities. For example the included intervention may: educate users about risk factors for HIV transmission; help users implement behavioural changes to reduce their risk for HIV acquisition or transmission; link users to local sexual health clinics; or support medication adherence. In addition, we selected only those interventions which utilize new technology and have the potential to be scaled and reach a large population. We therefore excluded interventions which utilize text (email or SMS) or recorded voice messaging as the only component(s) of the intervention, as well as interventions which use an in‐person component. We also excluded: conference proceedings; articles focused exclusively on formative information gathering (e.g. a paper describing focus group reactions to an intervention prototype would be included, but a focus group discussion of acceptability and preferences for a hypothetical intervention which has not yet been developed would not be included); and interventions for general health or generic support for managing any chronic disease.

From each article, we recorded intervention and study characteristics: the intervention name (if any), a brief description of the intervention, sample size, a brief description of the study, and study results. We also abstracted information about the target population for the intervention: HIV serostatus (HIV negative; HIV positive or both), age, gender, sexual orientation, race and ethnicity and substance use. We categorized each article (not mutually exclusive) based on the eHealth mode used (web‐based; smartphone app; social media; and/or game), the stage of the HIV care or prevention continua that was targeted (testing; education; behaviour change; linkage to sexual healthcare if HIV negative; PrEP care support; linkage to HIV care if HIV positive; and/or HIV care support), and the publication stage (early developmental; trial protocol; intervention‐only pilot trial; real‐world evaluation; or randomized controlled trial (RCT)). For interventions described in more than one publication, we assigned one publication stage based on the most advanced study design, in order from early developmental to RCT. Examples of interventions and their placement within the HIV treatment and prevention continua are shown in Figure 1.

We also searched the National Institutes of Health Research Portfolio Online Reporting Tools (NIH‐RePORT) for interventions still in the research pipeline. Our search, completed on 14 August 2018, included projects awarded funding in fiscal year(s) 2017 and/or 2018, as well as all other active projects. We used a more recent data range than the review of interventions in the published literature, in order to capture current trends in innovation by cataloguing projects with more recent funding. We used the same search strategy as used for peer‐reviewed publications. Project titles and abstracts were reviewed using the same inclusion and exclusion criteria for interventions in the published literature. Because these studies are ongoing, the interventions were not described in detail; therefore, we looked for basic summary information (often only 1 to 2 sentences) which indicated that development or testing of an intervention is a project aim, the intervention will utilize relevant new technology, and no indication of the exclusion criteria. To exclude interventions which were found in the published literature review, projects identified in the NIH‐RePORT were matched to the list of published interventions using the name of the intervention (if known), study author and project investigator names, project description, and any publications listed on the NIH‐RePORT Project Information Results. From the eligible project abstracts, we recorded the target population and the stage of the HIV prevention continuum that was targeted.

## 3 Results and discussion

The database search of published interventions yielded 2178 unique articles of which 203 were selected for full‐text review (Figure S1). After review, we excluded 90 articles: 14 lacked an HIV prevention endpoint; 26 used text or recorded voice messaging only; 23 required an in‐person component; and 27 lacked a clearly described intervention. We included 113 articles, describing 84 unique interventions for HIV prevention. The interventions are presented in Table 1, organized by eHealth mode of delivery and HIV serostatus population, with indication of the target point on the HIV care and prevention continua which the intervention addresses (additional intervention and study detail provided in Table S1).

**Table 1:** eHealth interventions found in the published literature, 2014 to 2018, organized by eHealth mode of delivery and HIV serostatus population, and categorized by the targeted point on the HIV care and prevention continua

Mode	Population	Citations	Intervention Name	Education^a^	Behaviour^a^	Testing^a^	Linkage^a^	Support^a^
App	HIV−	Browne et al. 20	mHealth Young Women’s CoOp (YWC)	•	•			
		Cordova et al. 21	Storytelling 4 Empowerment	•				
		Cordova et al. 22						
		Besoain et al. 23			•			
		Scott et al. 24			•			
		Goldenberg et al. 25	HealthMindr	•	•	•	•	•
		Goldenberg et al. 26						
		Sullivan et al. 27						
		Winskell et al. 28	Tumaini	•	•			
		Wray et al. 29	eTEST			•	•	
		Bauermeister et al. 12	Get Connected!	•	•	•	•	
		Bauermeister et al. 30						
		Horvath et al. 31						
		Yan et al. 32		•	•	•		
		Yang et al. 33	emocha	•	•			
	HIV+	Dillingham et al. 34	PositiveLinks				•	•
		Flickinger et al. 35						
		Flickinger et al. 36						
		Flickinger et al. 37						
		Dworkin et al. 38		•				•
		Himelhoch et al. 39	Heart2HAART					•
		Jacobs et al. 40						•
		Martin et al. 41	Care4Today					•
		Perera et al. 42						•
		Swendeman et al. 43		•	•			•
		Venter et al. 44	SmartLink				•	
		Westergaard et al. 45	mPeer2Peer					•
	HIV− or HIV+	Levy et al. 46				•	•	
		Ochalek et al. 47	HIV+Hepatitis Education	•				
		Reback et al. 48	Getting Off: Methamphetamine		•			
		Steinberg et al. 49	Teens in NYC			•	•	
Web‐based	HIV−	Ahmed‐Little et al. 50	RUClear			•		
		Bauermeister et al. 51	myDEx	•	•	•		
		Billings et al. 52	Safe Sistah	•	•			
		Brady et al. 53	TeensTalkHealth	•				
		Danielson et al. 54	SiHLEWeb	•	•			
		Fernandez et al. 55	POWER	•	•			
		Greene et al. 56	Keep It Up!	•	•	•		
		Motley et al. 57						
		Mustanski et al. 58						
		Mustanski et al. 59						
		Manavi et al. 60	Umbrella Health			•		
		Jones et al. 61	Guide‐Enhanced Love, Sex, and Choices	•	•			
		Kasatpibal et al. 62	rakplodpai.com	•	•			
		Klein et al. 63	Sexual Awareness for Everyone (C‐SAFE)	•	•			
		Maksut et al. 64		•	•	•		
		Mustanski et al. 65	Queer Sex Ed	•				
		Platteau et al. 66	Swab2Know	•		•		
		Loos et al. 67						
		Widman et al. 68	ProjectHeartforGirls.com	•				
		Wilson et al. 69	Sexual Health 24 (SH:24)			•	•	
		Wilson et al. 70						
		Ybarra et al. 71	CyberSenga	•	•			
	HIV+	Cote et al. 72	VIH‐TAVIE					•
		Cruess et al. 73	HINTS	•	•			
		Green et al. 74	HR‐VG	•	•			
		Hirshfield et al. 75	Sex Positive!	•	•			
		Horvath et al. 76	Thrive With Me	•				•
		Mi et al. 77		•	•	•	•	
		Milam et al. 78		•	•			
		Millard et al. 79	Positive Outlook					•
		Millard et al. 80						
		Miranda et al. 81	Condom‐HIM	•	•			
		Peterson et al. 82	CARE+ Corrections	•	•		•	•
	HIV− or HIV+	Anand et al. 83	Adam's Love	•		•	•	
		Anand et al. 84						
		Haas et al. 85	KNOW*NOW		•			
		Klein et al. 86	Real Talk	•	•			
		Lau et al. 87		•	•			
		Marsch et al. 88	Therapeutic Education System (TES)	•	•			
		Mitchell et al. 89	MCAP	•	•			
		Stephenson et al. 90	Project Nexus	•		•	•	
		Van den Berg et al. 91	Men2MenRI	•			•	
App & Web‐based	HIV−	de Boni et al. 92	A Hora É Agora	•		•	•	
		Dolwick Grieb et al. 93	¡Sólo Se Vive Una Vez!			•		
		Dolwick Grieb et al. 94						
		Stephenson et al. 95	Project Moxie			•	•	
	HIV+	Cho et al. 96	mobile Video Information Provider (mVIP)					•
		Cho et al. 97						
		Schnall et al. 98						
Social media	HIV−	Chiu et al. 99	HOPE	•		•		
		Garett et al. 100						
		Young et al. 101						
		Young et al. 102						
		Young et al. 103						
		Young et al. 104						
		Alarcon Gutierrez et al. 105				•		
		Bauermeister et al. 106	iREACH	•	•	•	•	
		Huang et al. 107	freehivselftests.weebly.com			•		
		Jenkins et al. 108		•				
		Lampkin et al. 109		•		•	•	
		Lelutiu‐Weinberger et al. 110	MiCHAT		•			
		Patel et al. 111	E‐PrEP	•				
		Rhodes et al. 112	CyBER			•		
		Sun et al. 113						
		Sun et al. 114		•	•			
		Tucker at al. 115		•		•		
		Tang et al. 116						
		Washington et al. 117	TIM Project	•		•		
	HIV+	Tanner et al. 118	weCARE				•	•
	HIV− or HIV+	Baltierra et al. 119	HealthMpowerment	•	•			
		Bauermeister et al. 120						
		Hightow‐Weidman et al. 121						
		Hightow‐Weidman et al. 122						
Game, app‐based	HIV+	Hightow‐Weidman et al. 123	AllyQuest	•	•			•
		LeGrand et al. 124	Epic Allies					•
		LeGrand et al. 125						
		Whiteley et al. 126	Battle Viro	•				•
Game, web‐based	HIV−	Shegog et al. 127	NATIVE‐It's Your Game	•				
		Schonnesson et al. 128	Project SMART	•	•			
		Fiellin et al. 11	PlayForward: Elm City Stories	•				
		Montanaro et al. 129						
		Fiellin et al. 130						
		Lukhele et al. 131	SwaziYolo	•				
		Enah et al. 132	Fast Car: Travelling Safely around the World	•				

a Target points on the HIV and care and prevention continua: Education; Behaviour change; Testing; Linkage to Care; Care Support.

**Figure 2:**
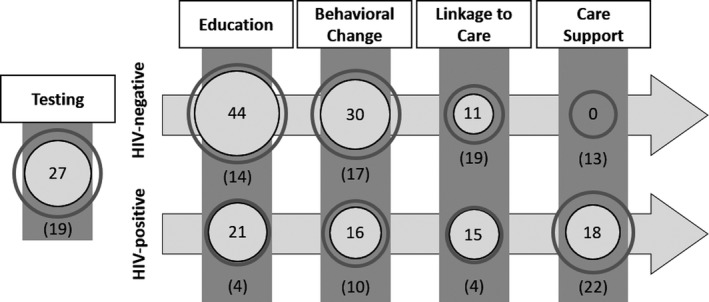
Number of interventions identified based on the targeted point of the HIV prevention continuum. eHealth interventions were found in a systematic review of peer reviewed literature (2014–2018). Categories are not mutually exclusive. Frequencies represent the number of interventions found in the published literature, with the area of the circles proportional to the number of interventions. The halos demonstrate how these circles would grow if projects recently awarded NIH funding are ultimately completed and published, with frequencies in parentheses to represent the number of projects with funding awarded. Linkage to Care and Care Support refer to PrEP care and HIV care for HIV‐negative and ‐positive populations, respectively.

We found 48 interventions (57%) designed solely for HIV‐negative users and 24 (29%) for only HIV‐positive users. Twelve (14%) additional interventions could be used by either HIV‐negative or HIV‐positive users. The distribution of the interventions along the care and prevention continua can be seen in Figure 2. Around one third of the interventions (n = 27; 32%) linked users to HIV testing services or served to adjunct home testing. Some of these testing interventions also provided the important bridge to HIV care for patients with positive results (n = 10; 37%) or to sexual health services for patients with negative results (n = 9; 33%). Among interventions for HIV‐negative users, most included an educational component (n = 44; 73%) and/or direct support for behaviour change (n = 30; 50%). Fewer facilitated linkage to clinical care (n = 11; 18%), where individualized counselling, routine HIV and STI screening, and access to biomedical interventions, such as PrEP, can occur. We found no interventions that were designed to support PrEP adherence or persistence. The interventions for HIV‐positive users were more evenly distributed along the prevention and care continua, including education (n = 21; 58%), behaviour change (n = 16; 44%), linkage to HIV care services (n = 15; 42%), and/or HIV care support (n = 18; 50%), including ART adherence.

The interventions were often designed for special populations, including MSM (n = 45; 54%), adolescents or young adults (n = 31; 37%), racial or ethnic minorities (n = 24; 29%), women (n = 7; 8%), transgender persons (n = 2; 2%), and/or persons who use drugs or alcohol (n = 8; 10%). Vulnerable or high‐risk groups, with intersectional identities and risk behaviours, were targeted with tailored interventions. Among the interventions for MSM, 13 (29%) targeted youth and 13 (29%) were for racial and ethnic minorities; four interventions (9%) were for young MSM of colour. Similarly, the 24 interventions for racial or ethnic minority populations were tailored for youth (n = 10; 42%) and women (n = 5; 21%). The majority (n = 71; 85%) of interventions were developed for users in resource rich countries. The remaining (n = 13; 15%) were intended to address the unique cultural needs of specific communities in low‐ or middle‐income countries.

Web‐based products, including traditional websites, messaging boards and modular content (e.g. interactive videos and quizzes), were the most common medium used for delivery of the interventions (n = 46; 55%), followed by apps (n = 33; 39%). We found eight interventions (10%) that communicated information using interactive gaming, including both web‐ and app‐based formats. Some of the interventions utilized existing social media platforms (n = 12; 14%) to reach large audiences with content, facilitate social support networks, and/or generate broad community discussion.

Most of the interventions (n = 58; 69%) were fully developed at the time of the most recent publication, with RCT (n = 28; 33%), intervention‐only pilot trial (n = 20; 24%) or real‐world evaluation (n = 10; 12%) results reported. The remaining interventions were still in early developmental stages (n = 10; 12%) or described in conjunction with a protocol for an ongoing or future RCT (n = 15; 18%). Among RCTs, the format for control or placebo conditions was widely variable. For example some studies used a standard‐of‐care or no‐intervention control condition, whereas others implemented a control condition designed to isolate the effects of either the intervention content or the mode of delivery. In studies testing the effect of the intervention content, placebo or general health content was delivered with technology comparable to the intervention (e.g. a game designed to improve HIV knowledge was compared to a control game that provided no health content) 11. Alternatively, to test the effects of new technologies, the control condition used traditional media formats to present content that was substantively the same or similar as the intervention (e.g. an individually tailored web intervention to connect users with relevant sexual health services was compared to a control condition that provided a simple sortable list of clinic locations) 12.

Using NIH‐RePORT, we identified 422 projects with funding in the years 2017 or 2018 or continued active support awarded in prior years. We removed 152 duplicate entries (consecutive annual funding for ongoing work), leaving 270 unique projects eligible for review. Of these, 81 projects met inclusion criteria: 19 were matched to the published literature review and excluded, leaving 62 interventions in the research pipeline.

We found 41 interventions (66%) in the research pipeline focused on HIV‐negative users and 28 (45%) for HIV‐positive users; seven (11%) of the interventions had features for both populations. Overall, 19 (31%) of the projects aim to promote or facilitate HIV testing. Among potential future interventions for HIV‐negative users, 14 (34%) will include educational content, 17 (41%) will promote behavioural modification, 9 (22%) will link users to clinical care, and 13 (29%) will support PrEP care. The interventions for HIV‐positive users will include components for education (n = 4; 14%), behavioural modification (n = 10; 36%), linkage to care (n = 4; 14%) and HIV care support (n = 22; 79%). Figure 2 shows how these interventions would contribute to the existing collection of interventions represented in the published literature.

Our systematic review found a robust collection of eHealth interventions, spanning important populations at risk for HIV, a balance of interventions addressing the prevention and care continua, and a wide variety of eHealth platforms. A major finding is that, for interventions in both the prevention and care continua, there is a greater representation of studies addressing more proximal steps in the continua than the distal steps. Thus, our review illustrates the need for more interventions focused on linkage to care, retention in care and adherence to therapies, especially PrEP.

Examination of funded studies that do not yet appear in published literature suggests that the next generation of eHealth interventions have a more expansive focus on distal phases of the continua. Notable among these are the 12 interventions to help users manage PrEP care. The importance of PrEP adherence has been well documented since at least 2012 13, 14, 15 and the National HIV/AIDS Strategy for the United States was updated in 2015 to prioritize comprehensive PrEP programmes including interventions for adherence 6. Interventions exist to address adherence to HIV treatment and these interventions may be useful for persons using PrEP as well. However, PrEP is used by individuals for prevention, rather than treatment, so there may be unique challenges to motivating adherence and persistence. The lack of any such eHealth interventions thus far in the published literature highlights the often slow process inherent to evidence‐based innovation. A recent review of smartphone app marketplaces found 11 PrEP interventions already available for download, including five with features for improving adherence, but quality was variable and none were found in the peer‐reviewed literature 16. These apps could be important stopgap tools, but a rigorous and scientific process is needed so that users and stakeholders can be confident that the tools work. Researchers can expedite the process with more efficient development, modification and testing protocols that do not always reinvent the wheel. A sustainable eHealth intervention will need to be flexible to meet new challenges in HIV care and prevention and transferrable to rapidly evolving eHealth technologies. One way to achieve this is to isolate and test the effects of individual components of an intervention thereby allowing future innovations to recycle and build upon effective strategies.

The overwhelming majority of interventions we found were designed for use in high‐income countries, where smartphones and high speed Internet are ubiquitous. It is important to note that our review excluded eHealth interventions that use only SMS or voice messaging. A recent systematic review of SMS and voice call interventions for HIV‐positive subjects found that a majority were for individuals in low‐ and middle‐income countries, contrasting the findings of this review 17. Limited access to new technologies in these settings has likely discouraged researchers from developing interventions that utilize these platforms. Consequently, innovative approaches to HIV prevention will not reach areas with the greatest global need. New technology interventions are not necessarily better than text and voice messaging interventions, and there remains a need for these interventions in geographies with poor technology infrastructure and in populations with low technology literacy. However, as technology becomes available to more people, equitable access to new technology interventions, if indeed superior, must be ensured. Comprehensive and country‐specific strategies should be implemented to maximize the benefit of currently available technologies and plan for future availability of others 18.

Our study has limitations typical of systematic reviews. We are limited in our electronic search to published articles, and there may be publication biases that lead to under‐representation of studies that have not been found to be efficacious; our analysis did not summarize efficacy, but we might have failed to enumerate studies for which the authors chose not to publish results or presented findings at a conference. We only searched two databases of published articles and our search terms, while selected to be comprehensive, may not be exhaustive. We have also missed early phase studies not yet published and funded through sources that would not appear in NIH‐RePORT – for example studies funded through foundational sources or through government health departments. Finally, our study excluded eHealth interventions that include other components (i.e. face‐to‐face counselling that is “boosted” by an app) which means we missed some interventions that fall within the new tech landscape. However, we chose to limit the scope of the review to interventions that can have broad impact.

We believe that there is value in periodically summarizing or updating the research pipeline based on the care and prevention continua and their elements. For example the Global HIV Vaccine Enterprise maintains a “pipeline project” with an updated description of the state of the vaccine field, from early concepts through randomized studies 19. Descriptions such as ours and the Global HIV Vaccine Enterprise document, allow funders and researchers to develop an understanding of relatively under‐investigated areas of study, and propose or fund projects to address important but understudied steps in the prevention and care continua.

## 4 Conclusions

The emergence of eHealth technologies has created opportunities for large‐scale dissemination of effective prevention interventions. This review provides an overview of recently published interventions as well as the next generation of interventions that are still in development. The research pipeline of eHealth interventions is robust, with a wide variety of knowledge, behavioural and care needs addressed. There is continued need for interventions to address adherence and retention in care for therapies, especially PrEP, service to key populations in low‐ and middle‐income countries, and development of more efficient and expedited processes for intervention research and development.

## Competing interests

KMM, AB, RW and PSS have no competing interests to declare.

## Authors’ contributions

KMM and PSS designed the study. KMM, AB and RW completed data collection and organized the data. All authors assisted with interpretation of results. KMM and PSS drafted the manuscript. All authors reviewed and agreed to submit the manuscript.

## Abbreviations

apps, smartphone applications; eHealth, mobile and other electronic technologies; MSM, men who have sex with men; NIH‐RePORT, National Institutes of Health Research Portfolio Online Reporting Tools; PrEP, pre‐exposure prophylaxis; RCT, randomized control trial; SMS, short message service; TW, transgender women.

## Supporting information


**Table S1.** eHealth interventions found in the published literature, 2014 to 2018

**Figure S1.** Results of the database search for recently published eHealth interventions.
