# Supplementary material for: Electronic and other new media technology interventions for HIV care and prevention: a systematic review
Source: J Int AIDS Soc. 2020 Jan 7;23(1):e25439. doi: 10.1002/jia2.25439 (PMC6945883; doi:10.1002/jia2.25439)
Supplement: Supplementary file 1 — Table S1. eHealth interventions found in the published literature, 2014 to 2018 [file JIA2-23-e25439-s002.docx]

**Electronic and other new media technology interventions for HIV prevention: a systematic review**

Authors: Kevin M. Maloney^1§^, Anna Bratcher^2^, Ryan Wilkerson^1^, Patrick S. Sullivan^1^

1 Department of Epidemiology, Emory University, Atlanta, GA, USA

2 Department of Epidemiology, University of California, Los Angeles, CA, USA

^§^ Corresponding author: Kevin M. Maloney
1520 Clifton Road
Atlanta, GA 30322, USA
Kevin.maloney@emory.edu

**Supplementary Table 1. eHealth interventions found in the published literature, 2014-2018**

| **eHealth mode** | **Target Population** | **Intervention Name** | **Sample Population; sample size; Location** | **Study design, intervention description, study results** | **Reference** |
| --- | --- | --- | --- | --- | --- |
| App | HIV- | mHealth Young Women’s CoOp (YWC) | African-American women, aged 18-25; target n = 700  North Carolina | RCT Protocol: The YWC app aims to reduce drug and alcohol use, sexual risk behaviors, and intimate partner violence, using videos to simulate in-person counseling. Women were randomized by county recruitment site to mHealth YWC, or a face-to-face counseling version of YWC, or to control. The trial is ongoing with completion of recruitment expected in 2019. | Browne et al. [1] |
| App | HIV- | Storytelling 4 Empowerment | Primarily African-American, aged 13-17; n = 30  Southeast Michigan | Pilot: Culturally appropriate and interactive stories to communicate information for sexual risk and drug use reduction using videos, graphics, and audio. Users found the application to have acceptable content, format, and process. A future trial will assess the intervention impact on HIV risk behaviors, HIV/STI testing, and drug use. | Cordova et al. [2]  Cordova et al. [3] |
| App | HIV- |  | MSM; n = 17  Barcelona, Spain | Pilot: a background smartphone application which displays HIV prevention messages when the user is seeking sex (via the web or other application) or is near a “hot zone” where sexual contacts are known to occur. The participants provided feedback to select ideal messaging for a future RCT. | Besoain et al. [4] |
| App | HIV- |  | Enrolled in substance use treatment, aged 18+; target n = 400  Chicago, IL | Research Protocol: RCT of a smartphone app based intervention utilizing ecological momentary assessments and interventions for ongoing self-monitoring and immediate recovery support. The primary outcome will be alcohol and substance use abstinence with secondary outcomes related to HIV risk behavior change. | Scott et al. [5] |
| App | HIV- | HealthMindr | MSM; n = 121  Atlanta, GA and Seattle, WA | Pilot: Usability and acceptability testing of a theory-based Android mobile phone app for HIV prevention, including education, commodity (condoms, HIV self-tests) ordering; screening for PrEP and nPEP; and prevention and treatment provider locators The intervention was acceptable to MSM and rated as having above-average usability. Most men used the commodity-ordering features of the app during the 4-month evaluation period, and nearly 1 in 10 PrEP-eligible men started PrEP, with most attributing their decision to start PrEP in part to the app. | Goldenberg et al. [6] Goldenberg et al. [7]  Sullivan et al. [8] |
| App | HIV- | Tumaini (“hope for the future”) | Aged 11-14 years; n = 60  Kisumu, Western Kenya | RCT: The Tumaini app uses role-playing, games, and a modular based curriculum to teach pre-adolescents about sexual health and HIV risk, and build risk reduction and avoidance skills. Participants were randomized to use the intervention for 16 days or control (no intervention). The intervention, used for an average of 27 hours by users in the intervention arm, was associated with increased sexual health knowledge, self-efficacy, intention to avoid risk behaviors, and intention to communicate sexual risk. | Winskell et al. [9] |
| App | HIV- | eTEST | MSM, aged 18+; interviews: n = 10; usability testing: n = 10.  United States | Pilot: Development and preliminary usability testing of a home based testing kit which utilizes Bluetooth and smartphone app connectivity to trigger follow-up telephone counseling and referral to services after a test kit has been opened. The eTEST system successfully detected test-kit use in 9/10 pilot cases. Participant perspectives also suggested that the timing, method (ie, phone call), and duration of follow-up were appropriate and helpful. | Wray et al. [10] |
| App | HIV- | Get Connected! | MSM, aged 15-24; target n = 480  United States | Research Protocol: RCT to receive tailored information, support and referral to local sexual health services, including PrEP and testing, based on individual needs; or to a control group to receive a comprehensive list of testing locations sortable by key characteristics. A pilot RCT (n = 130) found the intervention group were more likely to be tested for HIV/STIs than the control group (32% vs 22%, respectively) and were more likely to report using the information to change behaviors. The intervention has been refined and the primary outcomes of the ongoing trial are HIV/STI testing and PrEP awareness and willingness. | Bauermeister et al. [11]  Bauermeister et al. [12]  Horvath et al. [13] |
| App | HIV- |  | MSM, aged 16 and older; target n = 800.  Chagnsha, Hunan Province, China | Research Protocol: Participants will be randomized to a behavioral intervention mobile phone app or to usual care, to evaluate efficacy and cost-effectiveness. Results not yet available. | Yan et al. [14] |
| App | HIV- | emocha | African American MSM, aged 18+; n = 16  Baltimore, MD | Pilot: Feasibility and acceptability of a smartphone app to monitor alcohol use and related HIV-risk behaviors. Participants receive tailored messages based on recent behaviors. Participants felt the mobile technology was generally acceptable and provided feedback for improvements. | Yang et al. [15] |
| App | HIV+ | PositiveLinks | Newly diagnosed or at risk for falling out of care, aged ≥ 18 years; n = 77  Rural Southern United States | Pilot: Positive Links is a clinic-based online social support community with messaging boards, ART reminders and self-management, quizzes, and other content, designed to improve retention in care. Retention in care improved from 51% at baseline to 81% at 12 months; overall, mean CD4 count improved and the proportion of patients with a suppressed viral load increased from 47% to 79%. | Dillingham et al. [16]  Flickinger et al. [17]  Flickinger et al. [18]  Flickinger et al. [19] |
| App | HIV+ |  | African-American MSM, on ART for ≥ 3 months, aged 18-34; n = 16  Chicago, IL | Development: Iterative focus group study to improve a care support and retention intervention. The app uses notifications, reminders, and ART tracking, and uses a lifelike, talking avatar to guide users through aspects of HIV care, barriers to adherence, and socio-cultural issues relevant to young, African-American MSM living with HIV. Participants found the intervention to be acceptable and provided feedback for improvement, including issues related to privacy, stigma, and a desire for more positive feedback than negative. The next step for intervention development is pilot testing. | Dworkin et al. [20] |
| App | HIV+ | Heart2HAART | Substance use history and difficulty adhering to ART; n = 28  Baltimore, MD | Pilot RCT: Heart2HAART is a smartphone app designed to improve ART adherence through reminder messages. Participants randomized to Heart2HAART plus standard of care or standard of care. Patients reported being able to use the app without interference in their usual activities. There was no difference in adherence at 3 month follow-up in the intervention group compared to controls. | Himelhoch et al. [21] |
| App | HIV+ |  | Spanish speaking patients on ART; n = 25  Florida | Development: Mixed-methods study to adapt a successful English language intervention for Spanish speaking patients. The intervention is informative and provides suggestions for improving adherence. Results of the study informed the linguistic and cultural translation of the intervention for predominantly Spanish speaking patients. A future study will test the efficacy of the intervention. | Jacobs et al. [22] |
| App | HIV+ | Care4Today™ Mobile Health Manager | <95% recent ART adherence, aged 18+; n = 23  Southwest  United States | Pilot: Participants completed focus groups pre- and post- completion of a 3-month trial period utilizing the intervention. Care4Today™ assists ART adherence with scheduled reminders, weekly reports, and two-way provider messaging. Participants reported that the intervention was easy to use, helped organize their medication use, and provided a feeling of accountability. | Martin et al. [23] |
| App | HIV+ |  | Patients using ART; n = 28  Auckland, New Zealand | RCT: Participants randomized to the intervention group utilized an application with a built in medication timer and personalized visual representation of their disease management. The control group received an application with just the timer. The intervention was associated with greater ART adherence than the control group. The proportion of participants with a detectable viral load decreased (26% to 7%) in the intervention group, whereas the control group was associated with an increase in the proportion detectable (18% to 37%). | Perera et al. [24] |
| App | HIV+ |  | African American or Latino; n = 50  Los Angeles, CA | RCT: Biweekly self-management intervention for medication adherence, mental health, substance use, and sexual risk behaviors, compared to biweekly web-survey only. Participants described how self-monitoring functioned for self-expression to provide opportunities for catharsis through journaling, aspects of social support from non-judgmental disclosure, and in-the-moment availability, to improve their sense of well-being. | Swendeman et al. [25] |
| App | HIV+ | SmartLink | Newly diagnosed patients, aged ≥ 18 years; n = 353  South Africa | RCT Protocol: SmartLink provides CD4 and viral load testing results with information to help understand their meaning in order to keep patients engaged in care. Patients were randomized to SmartLink or to a standard of care arm. The manuscript describes the intervention development and trial process, with RCT results forthcoming. | Venter et al. [26] |
| App | HIV+ | mPeer2Peer | Past or current substance use, in care but not virally suppressed; n = 19  Baltimore, MD | Pilot: Development and acceptability of a smartphone application based intervention with personal care management and care navigator communication tools. In qualitative interviews, participants indicated that the peer navigation was acceptable and responded favorably to the smartphone application. | Westergaard et al. [27] |
| App | HIV- or HIV+ |  | Black MSM; n = 93  Washington, DC | Development: Participants were presented sample images from a smartphone app focused on improving access to culturally relevant HIV prevention and care services. Overall the participants reported interest in the intervention, would use it if available, and expected it to be effective. | Levy et al. [28] |
| App | HIV- or HIV+ | HIV+Hepatitis Education | Opioid use disorder and on wait-list for opioid agonist treatment, aged ≥ 18 years; n = 25  Vermont | Pilot: Part of a larger opioid agonist treatment trial, participants piloted the tablet intervention, which presents a flipbook on HIV basics and a video on hepatitis. Knowledge was assessed before and after the intervention, with feedback provided for incorrect answers. At baseline assessment, participants scored an average of 69% on HIV knowledge. After the intervention, the average score improved to 86%, with knowledge persisting at 4 and 12 week follow-up. | Ochalek et al. [29] |
| App | HIV- or HIV+ | Getting Off: Methamphetamine | Methamphetamine using MSM; n = 34  Los Angeles, CA | Pilot: Getting Off MA is an EMA monitoring app with web-based visualization dashboard, designed to support methamphetamine abuse treatment and associated sexual behavior. Participants were randomized to use the app with or without counselor support. Participants with (IRR = 0.02; 95% CI: 0.0, 0.3) and without (IRR = 0.23; 95% CI: 0.0, 3.6) counseling support both showed reduction in condomless anal sex, compared to historical controls. Neither group showed improvement in meth use compared to controls. | Reback et al. [30] |
| App | HIV- or HIV+ | Teens in NYC | Aged 12-19; n = 22,137 app downloads  New York City, NY | Evaluation: Developed from a paper-based resource, Teens in NYC is an application that locates sexual health care providers for adolescents and helps prepare users prior to a visit. Usage data was analyzed to determine patterns of app searches. From January 2013 to March 2016, the app was downloaded 22,137 times. Of 28,503 unique searches performed, 8,902 were for STI testing and treatment, 3,874 for HIV testing, and 849 for LGBTQ-specific services. | Steinberg et al. [31] |
| Web-based | HIV- | RUClear | ≥16 years old; n = 3,062  Greater Manchester, UK | Pilot: home HIV testing kits ordered online, with survey to assess acceptability and satisfaction. Of 3,062 tests, 7 new HIV infections were identified. The participants reported overall satisfaction with program. | Ahmed-Little et al. [32] |
| Web-based | HIV- | myDex | Single MSM, aged 18-24, reporting condomless anal sex; n = 180  United States | Research Protocol: Pilot RCT of a 6-session tailored program to educate YMSM about HIV risk reduction in same-sex dating and safer sex partner negotiation. myDEx uses interactive story-telling, case-scenarios, graphics, and videos. The trial is ongoing with 180 YMSM enrolled. Primary outcomes include number of risky sexual partnerships and HIV testing behavior at 30, 60, and 90 day follow-up. The results will inform a future efficacy trial. | Bauermeister et al. [33] |
| Web-based | HIV- | Safe Sistah | African-American women; n = 83  Washington, DC | RCT: behavioral intervention to increase HIV prevention behaviors versus delayed HIV education control arm. At 4-month follow up, women in the intervention arm reported using condoms for 59.5% of sexual acts (vs 44.2% at baseline) compared to 31.7% for women in the control arm (vs 39.1% at baseline). | Billings et al. [34] |
| Web-based | HIV- | TeensTalkHealth | Sexually active, aged 14-18; n = 147  Minneapolis, MN | Pilot: video, text-based, and other content, including discussion boards, with trained health educators, to promote safer sexual behaviors. Participants engaged with the website overall and found the content to be acceptable and useful. | Brady et al. [35] |
| Web-based | HIV- | SiHLEWeb | African-American women, aged 14-18; n = 18  San Francisco, CA | Pilot: module based risk-reduction program, modified from an effective in-person intervention, which increases knowledge and empowerment with culturally relevant content, including activities, games, and multimedia messaging. The website was considered an effective format for delivery of the intervention and participants provided feedback to improve technical aspects and content. | Danielson et al. [36] |
| Web-based | HIV- | POWER | Black, bisexual men; n = 224  Chicago, IL | RCT: Participants were randomized to an intervention to increase knowledge and decrease HIV risk behaviors with culturally relevant informative and motivational content or a standard health information control arm. Participants in the intervention group had reduced odds of condomless vaginal sex (OR = 0.49) or condomless anal sex (OR = 0.55) with male partners, compared to the control group, at 3 month follow up. | Fernandez et al. [37] |
| Web-based | HIV- | Keep It Up! | MSM, aged 18-29; n = 901  Chicago, IL | RCT: Keep It Up! is an online series of 7 modules, including games, videos, and interactive content, designed to increase HIV knowledge and improve safer-sex behavior. Participants were randomized to the intervention or to a control arm with 7 modules of static images and text. Participants in the intervention group showed a reduction in STIs (RR = 0.60; 95% CI: 0.38, 0.95) at 12 month follow up. Both arms of the trial showed a reduction in condomless anal sex with no difference between the groups. | Greene et al. [38]  Motley et al. [39]  Mustanski et al. [40]  Mustanski et al. [41] |
| Web-based | HIV- | Umbrella Health | n = 3099 test kits used  Birmingham and Solihull, UK | Evaluation: Home HIV and STI kits were available for online ordering by two municipal governments. Kits were tailored based on sex and sexual behavior. 5,310 test kits were ordered and 3,099 (58%) were returned. | Manavi et al. [42] |
| Web-based | HIV- | Guide-Enhanced Love, Sex, and Choices | Heterosexual women, aged 18 to 29; n = 40  Urban communities in MA and NJ, predominant-ly African-American, with high HIV prevalence | Pilot: A 12 episode soap opera video series which promotes safer-sex behaviors was adapted to include a peer video guide to prompt viewers to evaluate their own behaviors. At 30-day follow up, most (74%) of the women reported greater ease in discussing condoms with partners, while 44% had recently been tested for HIV. | Jones et al. [43] |
| Web-based | HIV- | rakplodpai.com | MSM; n = 162  Thailand | Pilot: Users were granted access to an informative HIV prevention website for 4 months and completed pre- and post- intervention behavior and knowledge questionnaires. Knowledge and safer-sex behavior scores improved after exposure to the website. | Kasatpibal et al. [44] |
| Web-based | HIV- | Sexual Awareness for Everyone (C-SAFE) | Latina women; n = 321  Southern CA and Orange County, FL | RCT: Women were randomized to a multimedia computer-delivered HIV prevention program tailored for Latinas, or to a control group, which received standard of care print-based messaging. The investigators found no significant differences in behavior or attitudes between the groups. | Klein et al. [45] |
| Web-based | HIV- |  | MSM, targeted but not exclusively black; n = 20  Atlanta, GA | Pilot: at home HIV testing with online video chatting and counseling sessions. A majority of participants found this method more favorable than testing in an office setting. | Maksut et al. [46] |
| Web-based | HIV- | Queer Sex Ed | In same-sex relationship, aged 16-20; n = 202  United States | Pilot: 5-module online multimedia intervention to increase safer sex knowledge and improve relationship communication skills and self-acceptance. The study showed that Queer Sex Ed is feasible and acceptable. It also showed significant increases in 15 of 17 tested outcomes | Mustanski et al. [47] |
| Web-based | HIV- | Swab2Know | MSM or sub-Saharan African migrants; n = 289  Belgium | Evaluation: HIV-testing program within a website providing information, prevention messages, and test results  430 tests from 289 participants were ordered. Self-sampling procedures were of acceptable quality and users were highly satisfied with the project. | Platteau et al. [48]  Loos et al. [49] |
| Web-based | HIV- | ProjectHeartforGirls.com | Ethnically-diverse women, aged 16-19; focus groups: n = 25; usability testing: n = 5  North Carolina | Development: An interactive and navigable web-based program, with animated videos and modules to improve sexual communication skills and reduce HIV/STDS among adolescent girls. The intervention was developed and refined with community input. The intervention was found to be generally acceptable and easy to navigate. Feasibility, acceptability, and preliminary efficacy testing is currently being conducted. | Widman et al. [50] |
| Web-based | HIV- | Sexual Health 24 (SH:24) | Sexually active, aged 16-30; n = 2,072  London, UK | RCT: Participants were randomized to receive access to home HIV/STI testing or to a control group with access to a list of local sexual health clinics. Participants in the intervention group were more likely to receive testing (50% vs 27%) compared to the control group; relative risk = 1.9 (95% CI: 1.6, 2.2). | Wilson et al. [51]  Wilson et al. [52] |
| Web-based | HIV- | CyberSenga | Students, aged 9-11; n = 366  Mbarara, Uganda | RCT: Students were randomized to an internet-based 5-module knowledge and behavior intervention or to a control arm.  Participants’ HIV-related knowledge improved over time at a greater rate for the intervention groups compared to the control group. There was no difference between the groups in abstinence behaviors, but the intervention group was more likely to endorse condom use motivation. | Ybarra et al. [53] |
| Web-based | HIV+ | HIV Treatment,  Virtual Nursing Assistance and Education (VIH-TAVIE) | Patients engaged in care; n = 179  Canada | RCT: A quasi-experimental study (non-randomized) of the VIH-TAVIE intervention, a tailored online program to manage HIV symptoms and support ART adherence, compared to standard of care nursing follow-up. Adherence improved at 6-month follow-up for patients in both treatment groups. No statistically significant difference between the groups was observed. | Cote et al. [54] |
| Web-based | HIV+ | HIV Internet Sex (HINTS) | MSM reporting CAS, aged 18+; n = 167  United States | RCT: The HINTS intervention is a 4-session online facilitated support group designed to increase safer sex behaviors. Men randomized to the control group participated in similar group discussions except with nonsexual general health content. There were no significant differences between groups in condomless anal sex with all partners at 6 month follow-up. Men in the intervention group reported a lower rate of condomless anal sex with HIV-negative or serostatus unknown partners compared to the control group (IRR: 0.37; 95% CI: 0.21-0.67). | Cruess et al. [55] |
| Web-based | HIV+ | Healthy Relationships Video Group (HR-VG) | Sexually active women; n = 71  United States | RCT: Women were randomized to HR-VG or to a control group. HR-VG is a six session online video support group to help women with safer sex and serostatus disclosure. The intervention has previously been found to be effective. A subsample of participants (n = 21) in this qualitative study reported their preferences for using HR-VG at home versus a community based organization. The women emphasized the need for privacy. | Green et al. [56] |
| Web-based | HIV+ | Sex Positive! | MSM, with poor ART adherence or a detectable viral load and recent CAI with HIV- or serostatus unknown partner(s), aged 18 or older; target n = 1500  United States | Research Protocol: Ten web-based video vignettes designed to decrease CAI and increase serostatus disclosure will be delivered to men randomized to intervention versus general health videos presented to the control group. The study has not yet been implemented. Previously published pilot studies of the intervention have suggested efficacy. | Hirshfield et al. [57] |
| Web-based | HIV+ | Thrive With Me | MSM, with sub-optimal ART adherence; target n = 400 (50% enrollment target for substance using MSM)  New York City, NY | RCT Protocol: Thrive With Me provides peer support through a private asynchronous social network, and multimedia content for tailored HIV care information, medication reminders, and self-monitoring. Subjects will be randomized to receive the intervention or control (weekly newsletter) for 5 months.  The trial will follow participants for 17 months. Enrollment completed in April 2018 and follow-up will continue through August 2019. The primary outcome will be viral load and viral suppression. Secondary outcomes include self-reported ART adherence, engagement in HIV care, and transmission risk behaviors. | Horvath et al. [58] |
| Web-based | HIV+ |  | MSM, aged 18+; n = 202  Chengdu, China | RCT: participants were randomized to a four module intervention (information, messaging boards, counseling, and interactive game) for serostatus disclosure, encouraging partners to test for HIV, sexual risk reduction, and ART initiation, or to a standard of care control group. The intervention group were more likely to disclose HIV status to their partners (76.0% versus 61.2%) and to motivate partners to seek HIV testing (42.3% vs. 25.5%). | Mi et al. [59] |
| Web-based | HIV+ |  | MSM in care; n = 179  Southern California | RCT: participants were randomized to receive monthly risk reduction messages tailored to their recently self-reported behaviors or to a control arm which received no intervention. No difference in 12 month STI incidence, condomless sex and disclosure of HIV status between intervention and control arm. | Milam et al. [60] |
| Web-based | HIV+ | Positive Outlook | MSM, aged 18+; feasibility pilot study: n = 10; RCT: n = 37  Australia | Pilot and RCT: a pilot study to assess the feasibility and preliminary efficacy of a web-based self-management program to address the psychosocial impact of living with HIV; participants in the control arm of the RCT received standard of care. The program was well accepted, but showed a decline in engagement over the duration of the study. Those who used the program showed increases in HIV-related quality of life, self-efficacy, self-management skills, social support and adjustment to HIV. | Millard et al. [61]  Millard et al. [62] |
| Web-based | HIV+ | Condom-HIM | MSM  Canada | Development: individually tailored web-based intervention to increase condom use. Results not yet available. | Miranda et al. [63] |
| Web-based | HIV+ | CARE+ Corrections | Recently released from a correctional facility, aged ≥ 18 years; n = 24  Rhode Island and Washington, DC | Pilot: The intervention provides computer-based counseling and skill building for HIV transmission risk reduction planning, ART adherence, and transition to HIV care after release, as well as automated SMS messages for care support after release. In qualitative interviews, the participants reported that the intervention was feasible and acceptable for use before and after re-integration to communities after release from correctional facilities. | Peterson et al. [64] |
| Web-based | HIV- or HIV+ | Adam's Love | MSM & TW  Thailand | Evaluation: a web-based program with built in social media to provide information about HIV prevention, testing, and treatment, including real-time online counseling and direct scheduling of testing. 11,120 MSM received counseling, 8,288 linked to testing, 1,112 HIV+ MSM advised on treatment. | Anand et al. [65]  Anand et al. [66] |
| Web-based | HIV- or HIV+ | KNOW*NOW | MSM: n = 8; HIV- MSM: n = 8; HIV- heterosexuals: n = 12  Midwest, United States | Pilot: Focus group discussions ascertained acceptability of a web-based clinic-linked portal displaying HIV related information, including test results, PrEP use, and viral suppression, to facilitate communication with sexual partners. Participants were followed for 6-months for utilization of the service. Regardless of sexual orientation, HIV-negative participants were generally interested in using the service, whereas the HIV-positive MSM were mixed in their opinions and interest, due to stigma and privacy. During follow-up, 94% of participants accessed the portal and 69% reported showing it to a sexual partner. | Haas et al. [67] |
| Web-based | HIV- or HIV+ | Real Talk | Black MSM; n = 226  Florida, New Jersey, and Georgia | RCT: Men were randomized to a module based intervention program or to read sexual health brochures. The intervention uses culturally relevant and affirming audio narration, activities, games, video and visual content presentations for HIV risk reduction among black HIV- and HIV+ MSM. Men in the intervention group were more likely to express intentions to engage in safer sex behaviors, but there were no differences between groups in actual condom use and other harm reduction behaviors. | Klein et al. [68] |
| Web-based | HIV- or HIV+ |  | Sexually active MSM, aged 18+; n = 402  Hong Kong, China | RCT: Participants were randomized to view one of three online interventions: informative videos; informative videos plus fear-based content; or basic HIV related information. Fewer subjects reported CAS with a casual partner at 3 month follow-up compared to baseline (videos: 11.7% vs. 19.1%; videos plus fear based content: 11.9% vs. 22.9%; basic information: 12.7% vs. 19.6%). There were no between group differences observed. | Lau et al. [69] |
| Web-based | HIV- or HIV+ | Therapeutic Education System (TES) | New patients at outpatient substance abuse treatment, aged 12-18; n = 141  New York City, NY | RCT: participants were randomized to a web-based intervention for HIV education, including information about treatment and prevention, or to a traditional educator-delivered control program. Both arms of the study experienced favorable outcomes, demonstrating improvements in STI knowledge and related skills, as well as reductions in risk behaviors. The web-based and educator-delivered interventions were equally effective. | Marsch et al. [70] |
| Web-based | HIV- or HIV+ | Male Couples Agreement Project (MCAP) | HIV-positive MSM couples: n = 8 couples; HIV-serodiscordant MSM couples: n = 10 couples  Miami-Fort Lauderdale metro area, Florida | Development: Pilot study with qualitative interviews to adapt a toolkit for facilitating sexual agreements in HIV-negative MSM couples for use by HIV-positive and HIV-serodiscordant MSM couples. Participants suggested that the intervention be refined to include biomedical prevention modalities (e.g. PrEP) and other ways to protect each other from HIV/STIs. The participants also wanted the intervention to address other important health issues, such as mental health, exercise, and nutrition. | Mitchell et al. [71] |
| Web-based | HIV- or HIV+ | Project Nexus | Sero-concordant-negative couples; target n = 200 dyads. Sero-discordant couples; target n = 200 dyads. 219 couples enrolled to date.  United States | Research Protocol: RCT of home HIV-testing with video counseling intervention compared to a control group of HIV-testing only. The counseling is designed to improve couples' management of HIV risk, formation and adherence to explicit sexual agreements, and sexual risk-taking. Preliminary data: 88% control couples have reported their home HIV testing results; 76.6% intervention couples have scheduled and completed the video-chat counseling session; 5.9% of all tests were preliminary positive, of which 73% were linked to care. | Stephenson et al. [72] |
| Web-based | HIV- or HIV+ | Men2MenRI | Black and Hispanic MSM, aged ≥ 18 years  Providence metropolitan area, RI and MA | Development Protocol: Adaptation of an existing website (Men2MenRI, originally designed for white MSM) to be more culturally relevant for black and Hispanic MSM. The new interactive website will focus on PrEP and TasP, in addition to behavioral information. The website will be refined using qualitative methods and an open-pilot, followed by a small RCT. The project is expected to continue until August 2020. The RCT will assess change in sexual risk behaviors, knowledge and uptake of PrEP and TasP. | Van den Berg et al. [73] |
| App & Web-based | HIV- | A Hora É Agora | MSM, aged ≥ 18 years; n = 23,878  Curitiba, Brazil | Evaluation: Program evaluation of a multi-pronged community intervention, delivered by a web-based platform and mobile app to increase HIV knowledge, evaluate risk, and distribute HIV self-test kits. Over two years, the intervention was accessed by 23,878 unique users and 7,352 self-test kits were ordered. The system did not require users to report results, although 34 individuals reported a positive test. At an affiliated clinic, 44 users obtained confirmatory testing and 40 were linked to HIV care services. | de Boni et al. [74] |
| App & Web-based | HIV- | ¡Sólo Se Vive Una Vez! | Foreign born Latino/Hispanic men; n = 104  Baltimore, MD | Pilot: Participants completed a survey to identify barriers to testing (e.g. fear of diagnosis) which they may be experiencing. Based on the results, participants were presented relevant video modules addressing the barrier. After the intervention, there was a statistically significant increase in intention to test for HIV within 3 months. | Dolwick Grieb et al. [75]  Dolwick Grieb et al. [76] |
| App & Web-based | HIV- | Project Moxie | Transgender or gender non-conforming, aged 15-24; target n = 200  United States | Research Protocol: RCT to receive a home HIV test plus a video-chat counseling intervention or home HIV test alone. The primary outcome will be HIV testing completion with secondary outcomes related to HIV risk behaviors, linkage to HIV care (if test positive) and intervention acceptability. | Stephenson et al. [77] |
| App & Web-based | HIV+ | mobile Video Information Provider (mVIP) | Low-income with recent symptoms, aged 18+; n = 80  New York City, NY | RCT: All participants received the mVIP app for HIV symptom assessment. Participants randomized to the intervention group were also presented tailored self-care strategies. Improvement in ART adherence, a secondary outcome of the study, was dependent on the scale used to measure adherence: one scale showed statistically greater improvement in the intervention group compared to the control group while another scale showed no difference. | Cho et al. [78]  Cho et al. [79]  Schnall et al. [80] |
| Social media | HIV- | HOPE Harnessing Online Peer Education (HOPE) | MSM, aged 18+; n = 556  Lima, Peru | RCT: Participants were randomized to join a Facebook group and interact with trained peer leaders (intervention) or to receive standard health information and participate in a Facebook group without peer leaders (control) to test the efficacy of promoting HIV testing. In the Peru study, the intervention group had 2.79 times the odds of requesting an HIV test and 2.61 times the odds of getting tested for HIV, compared to the control group. In California, a greater proportion of intervention participants (44%) compared to control participants (20%) requested a test kit. | Chiu et al. [81]  Garett et al. [82]  Young et al. [83]  Young et al. [84]  Young et al. [85]  Young et al. [86] |
|  |  |  | African American or Latino MSM, aged 18+; n = 112  Los Angeles, CA |  |  |
| Social media | HIV- |  | MSM; n = 4,283 app users contacted  Barcelona, Spain | Evaluation: Pilot study of an outreach program for HIV, STI, and hepatitis C testing; geosocial-sexual networking app users were sent unsolicited messages and invited to a local facility for testing. 1,019 users responded to messages with 846 responding favorably. One new HIV infection was identified among 79 men tested; 45% had not been tested within the past year and 8% had never been tested. | Alarcon Gutierrez et al. [87] |
| Social media | HIV- | iREACH | MSM, aged 13-18; target n = 600  United States | Research Protocol: RCT for efficacy of iREACH, a tailored application, with educational modules, goal-setting, connection to local resources, and peer video support, designed to reduce HIV vulnerability with life skills support. The trial is currently recruiting participants. Primary outcomes are related to ability and intentions to use HIV prevention strategies as well as HIV/STI testing. Secondary outcomes include actual individual behaviors. | Bauermeister et al. [88] |
| Social media | HIV- | freehivselftests.weebly.com | Black or Latino MSM, aged 18 or older; n = 300,000 banner advertisement views  West Hollywood and downtown LA, CA | Evaluation: A location-based social/sexual networking smartphone application was used to advertise a home HIV self-test kit available through online ordering. Follow up surveys were distributed to evaluate the program. 11,939 unique website visits and 334 test kits ordered; 122 men completed surveys with 68% reporting preference from home testing and 2 reporting a positive result. | Huang et al. [89] |
| Social media | HIV- |  | MSM; n = 673  North-central North Carolina | Evaluation: A profile was created for a health educator on four location-based social/sexual networking smartphone apps to allow users to ask questions about sexual health and local resources; conversations were evaluated qualitatively. The perception of authenticity and confidentiality helped build trust between the health educator and users, facilitating the sharing of information. | Jenkins et al. [90] |
| Social media | HIV- |  | MSM; estimated population of n = 16,657  Suburban San Mateo County, CA | Evaluation: In a setting with no physical venues catering to the MSM community, outreach health educators used location-based social/sexual networking smartphone apps to provide education, promote testing, and link MSM to care. Implementation of the program increased health department engagement with the MSM community by 1500%. MSM received information and 14 were tested for BSTI and HIV, with one new HIV infection identified. | Lampkin et al. [91] |
| Social media | HIV- | Motivational Interviewing (MI) Communication about Health, Attitudes, and Thoughts (MiCHAT) | Substance using MSM, aged 18-29; n = 41  United States | Pilot: A successful in-person intervention was adapted for use online with Facebook chat. The intervention uses up to 8 one-hour live chat sessions with a trained counselor. Participants reported decreased HIV risk behaviors, including condomless anal sex and condomless anal sex while using substances, in post-intervention surveys compared to pre-intervention. | Lelutiu-Weinberger et al. [92] |
| Social media | HIV- | E-PrEP: Empowering with PrEP | Black or Latinx MSM, aged 18-29; N = 10 peer leader; n = 152 network participants  New York City, NY | Research Protocol: Cluster randomized trial of a social media intervention to increase PrEP uptake. Peer leaders randomly assigned to promote either E-PrEP or control (general health messaging) on social media. Network contacts were enrolled and followed for change in intention to use PrEP. The study was completed in November 2017 with results still being analyzed. | Patel et al. [93] |
| Social media | HIV- | CyBER | MSM; n = 1292  United States | RCT: Four communities randomized to receive an intervention (N = 2) promoting HIV testing on social and sexual networking websites or no intervention (N = 2). Intervention communities showed higher proportion testing for HIV (64%) than control communities (42%) post-intervention, with no difference pre-intervention (35% vs 39%, respectively). | Rhodes et al. [94]  Sun et al. [95] |
| Social media | HIV- |  | College students; n = 196  Hong Kong, China | RCT: Participants were randomized to join an intervention Facebook group or to a control group accessing a sexual health website. Peer educators posted content to the group and moderated discussion. Participants in the intervention group but not control group showed increased condom use attitudes; there were no between group differences. The intervention was feasible and effective, but not more effective than the control. | Sun et al. [96] |
| Social media | HIV- |  | MSM, aged ≥ 16 years; n = 1,381  China | RCT: Stepped-wedge RCT of 8 cities randomized to implement a crowdsourced intervention for HIV testing at different times. The intervention was developed through a social media contest for MSM to submit promotional materials. Participants were followed for HIV test uptake pre- and post- intervention. After introduction of the intervention, a greater proportion (8.9%; 95% CI: 2.2%-15.5%) of participants reported testing for HIV, with 48.6% of participants overall reporting testing. | Tucker at al. [97]  Tang et al. [98] |
| Social media | HIV- | TIM Project | HIV status unknown with no HIV test within past 6 months, BMSM, aged 18-30;  n = 42  Los Angeles, CA | RCT: BMSM randomized to receive weekly videos promoting HIV testing and risk reduction in a Facebook group or to a control Facebook group showing similar content with text only. Participants were encouraged to comment and discuss. At 6 weeks follow-up, BMSM in the intervention group had 7-times greater odds (95% CI: 1.7, 28.3) of testing for HIV, compared to the control group, and showed greater improvement in HIV knowledge. | Washington et al. [99] |
| Social media | HIV+ | weCARE | MSM, aged 13-35  United States | Development: Theory and design process for a social media based intervention to improve HIV related outcomes at every stage of the continuum. Results not yet available. | Tanner et al. [100] |
| Social media | HIV- or HIV+ | HealthMpowerment | Black men and transgender women who have sex with men, aged 18-30; n = 474  Central North Carolina | RCT: A virtual community of HIV-negative and HIV-positive YBMSM and TW, designed to reduce sexual risk behaviors and decrease stigma, through social support and multimedia content. Participants randomized to the control received information only. A supportive and engaged virtual community was established, with participants showing positive social support and improved mental health indicators. Participants in the intervention arm reported a reduced rate of condomless anal sex (0.76, 95% CI: 0.69,0.83) compared to the control arm. | Baltierra et al. [101]  Bauermeister et al. [102]  Hightow-Weidman et al. [103]  Hightow-Weidman et al. [104] |
| Game, app-based | HIV+ | AllyQuest | MSM, aged 19-24; n = 20  Chicago, IL | Pilot: A smartphone game app to improve HIV care self-management and risk behaviors using a medication tracker, participatory storytelling, activities, discussion boards, multimedia messages, and reinforcing rewards and achievements. The YMSM found the game to be acceptable and easy to use. Greater engagement with the app was associated with reporting positive care outcomes and self-confidence in medication adherence. | Hightow-Weidman et al. [105] |
| Game, app-based | HIV+ | Epic Allies | MSM, aged 18-29; focus groups: n = 20; usability testing: n = 7; RCT: target n = 146  North Carolina | Development and Research Protocol: RCT protocol for a smartphone based game designed to enhance ART adherence following development and pilot testing. The final prototype received favorable usability scores from participants. An RCT has been completed and results are forthcoming. | LeGrand et al. [106]  LeGrand et al. [107] |
| Game, app-based | HIV+ | Battle Viro | Aged 18-26; n = 20  Rhode Island | Development: Battle Viro is an interactive role-playing game to improve knowledge, motivation, and skills for HIV care management.  Participants informed the iterative development of the game, from concept to prototype, through qualitative interviews and quantitative measurements of usability of the prototype. | Whiteley et al. [108] |
| Game, app-based | HIV+ | NATIVE-It's Your Game | American Indian/Alaska Native students, aged 9-16  Arizona, Arkansas, Pacific Northwest | Development: Internet-based HIV/STI and pregnancy prevention curriculum. Tribal stakeholders approved of the intervention, and determined it to be culturally appropriate for AI/AN youth, and acceptable for implementation in tribal settings. | Shegog et al. [109] |
| Game, app-based | HIV+ | Project SMART | HIV-negative MSM, aged 15+; n = 58  Sweden | RCT: Men were randomized to the intervention or to a waitlist control group. Project SMART is a modular, role-playing game designed to reduce sexual risk behavior. The intervention was associated with improved HIV knowledge and intention to use condoms. | Schonnesson et al. [110] |
| Game, app-based | HIV+ | PlayForward: Elm City Stories | Aged 11-14; n = 333  New Haven, CT | RCT: participants were randomized to an intervention game aimed at increasing knowledge around substance use and the associated risk factors for HIV or to a set of attention- and time-control games. The study found no differences in initiation of sexual activity (low in both groups) but adolescents in the intervention group showed a greater increase in knowledge and improvement in attitudes toward sexual health. | Fiellin et al. [111]  Montanaro et al. [112]  Fiellin et al. [113] |
| Game, app-based | HIV+ | SwaziYolo | Aged 18-29 years old; n = 380  Swaziland | Research Protocol: a two-arm randomized intervention trial with an interactive, educational story game intervention and a wait-list control. Results expected Feb 2017. | Lukhele et al. [114] |
| Game, app-based | HIV+ | Fast Car: Travelling Safely around the World | African-American, aged 12-16; n = 42  Rural Alabama | Development: Focus group study of acceptability and relevance of the Fast Car game available from UNESCO. Participants found the game concept to be desirable for communicating information about HIV, but did not find the Fast Car intervention to be engaging and desired improvements in the game’s functionality as well as more relevant and individualized content. | Enah et al. [115] |

Citations

1. Browne, F.A., et al., *mHealth versus face-to-face: study protocol for a randomized trial to test a gender-focused intervention for young African American women at risk for HIV in North Carolina.* BMC Public Health, 2018. **18**(1): p. 982.

2. Cordova, D., et al., *The Usability and Acceptability of an Adolescent mHealth HIV/STI and Drug Abuse Preventive Intervention in Primary Care.* Behav Med, 2016: p. 1-12.

3. Cordova, D., et al., *A Community-Engaged Approach to Developing an mHealth HIV/STI and Drug Abuse Preventive Intervention for Primary Care: A Qualitative Study.* JMIR Mhealth Uhealth, 2015. **3**(4): p. e106.

4. Besoain, F., et al., *Prevention of sexually transmitted infections using mobile devices and ubiquitous computing.* Int J Health Geogr, 2015. **14**: p. 18.

5. Scott, C.K., M.L. Dennis, and D.H. Gustafson, *Using smartphones to decrease substance use via self-monitoring and recovery support: study protocol for a randomized control trial.* Trials, 2017. **18**(1): p. 374.

6. Goldenberg, T., et al., *Preferences for a Mobile HIV Prevention App for Men Who Have Sex With Men.* JMIR Mhealth Uhealth, 2014. **2**(4): p. e47.

7. Goldenberg, T., et al., *Building a Mobile HIV Prevention App for Men Who Have Sex With Men: An Iterative and Community-Driven Process.* JMIR Public Health Surveill, 2015. **1**(2): p. e18.

8. Sullivan, P.S., et al., *Usability and Acceptability of a Mobile Comprehensive HIV Prevention App for Men Who Have Sex With Men: A Pilot Study.* JMIR Mhealth Uhealth, 2017. **5**(3): p. e26.

9. Winskell, K., et al., *A Smartphone Game-Based Intervention (Tumaini) to Prevent HIV Among Young Africans: Pilot Randomized Controlled Trial.* JMIR Mhealth Uhealth, 2018. **6**(8): p. e10482.

10. Wray, T., et al., *eTEST: Developing a Smart Home HIV Testing Kit that Enables Active, Real-Time Follow-Up and Referral After Testing.* JMIR Mhealth Uhealth, 2017. **5**(5): p. e62.

11. Bauermeister, J.A., et al., *Acceptability and preliminary efficacy of a tailored online HIV/STI testing intervention for young men who have sex with men: the Get Connected! program.* AIDS Behav, 2015. **19**(10): p. 1860-74.

12. Bauermeister, J.A., et al., *A Multilevel Tailored Web App-Based Intervention for Linking Young Men Who Have Sex With Men to Quality Care (Get Connected): Protocol for a Randomized Controlled Trial.* JMIR Res Protoc, 2018. **7**(8): p. e10444.

13. Horvath, K.J. and J.A. Bauermeister, *eHealth Literacy and Intervention Tailoring Impacts the Acceptability of a HIV/STI Testing Intervention and Sexual Decision Making Among Young Gay and Bisexual Men.* AIDS Educ Prev, 2017. **29**(1): p. 14-23.

14. Yan, J., et al., *Development and effectiveness of a mobile phone application conducting health behavioral intervention among men who have sex with men, a randomized controlled trial: study protocol.* BMC Public Health, 2017. **17**(1): p. 355.

15. Yang, C., et al., *Feasibility and Acceptability of Smartphone-Based Ecological Momentary Assessment of Alcohol Use Among African American Men Who Have Sex With Men in Baltimore.* JMIR Mhealth Uhealth, 2015. **3**(2): p. e67.

16. Dillingham, R., et al., *PositiveLinks: A Mobile Health Intervention for Retention in HIV Care and Clinical Outcomes with 12-Month Follow-Up.* AIDS Patient Care STDS, 2018. **32**(6): p. 241-250.

17. Flickinger, T.E., et al., *Social Support in a Virtual Community: Analysis of a Clinic-Affiliated Online Support Group for Persons Living with HIV/AIDS.* AIDS Behav, 2017. **21**(11): p. 3087-3099.

18. Flickinger, T.E., et al., *Content Analysis and User Characteristics of a Smartphone-Based Online Support Group for People Living with HIV.* Telemed J E Health, 2016. **22**(9): p. 746-54.

19. Flickinger, T.E., et al., *Addressing Stigma Through a Virtual Community for People Living with HIV: A Mixed Methods Study of the PositiveLinks Mobile Health Intervention.* AIDS Behav, 2018.

20. Dworkin, M., et al., *A Realistic Talking Human Embodied Agent Mobile Phone Intervention to Promote HIV Medication Adherence and Retention in Care in Young HIV-Positive African American Men Who Have Sex With Men: Qualitative Study.* JMIR Mhealth Uhealth, 2018. **6**(7): p. e10211.

21. Himelhoch, S., et al., *Pilot feasibility study of Heart2HAART: a smartphone application to assist with adherence among substance users living with HIV.* AIDS Care, 2017. **29**(7): p. 898-904.

22. Jacobs, R.J., et al., *Development of a culturally appropriate computer-delivered tailored Internet-based health literacy intervention for Spanish-dominant Hispanics living with HIV.* BMC Med Inform Decis Mak, 2014. **14**: p. 103.

23. Martin, C.A. and M.J. Upvall, *A Mobile Phone HIV Medication Adherence Intervention: Acceptability and Feasibility Study.* J Assoc Nurses AIDS Care, 2016. **27**(6): p. 804-816.

24. Perera, A.I., et al., *Effect of a smartphone application incorporating personalized health-related imagery on adherence to antiretroviral therapy: a randomized clinical trial.* AIDS Patient Care STDS, 2014. **28**(11): p. 579-86.

25. Swendeman, D., et al., *Smartphone self-monitoring to support self-management among people living with HIV: perceived benefits and theory of change from a mixed-methods randomized pilot study.* J Acquir Immune Defic Syndr, 2015. **69 Suppl 1**: p. S80-91.

26. Venter, W., et al., *Improving Linkage to HIV Care Through Mobile Phone Apps: Randomized Controlled Trial.* JMIR Mhealth Uhealth, 2018. **6**(7): p. e155.

27. Westergaard, R.P., et al., *Acceptability of a mobile health intervention to enhance HIV care coordination for patients with substance use disorders.* Addict Sci Clin Pract, 2017. **12**(1): p. 11.

28. Levy, M.E., et al., *Acceptability of a Mobile Smartphone Application Intervention to Improve Access to HIV Prevention and Care Services for Black Men Who Have Sex with Men in the District of Columbia.* Digit Cult Educ, 2015. **7**(2): p. 169-191.

29. Ochalek, T.A., et al., *A novel mHealth application for improving HIV and Hepatitis C knowledge in individuals with opioid use disorder: A pilot study.* Drug Alcohol Depend, 2018. **190**: p. 224-228.

30. Reback, C.J., et al., *Ecological momentary assessments for self-monitoring and counseling to optimize methamphetamine treatment and sexual risk reduction outcomes among gay and bisexual men.* J Subst Abuse Treat, 2018. **92**: p. 17-26.

31. Steinberg, A., et al., *Evaluation of a Mobile Phone App for Providing Adolescents With Sexual and Reproductive Health Information, New York City, 2013-2016.* Public Health Reports, 2018. **133**(3): p. 234-239.

32. Ahmed-Little, Y., et al., *Attitudes towards HIV testing via home-sampling kits ordered online (RUClear pilots 2011-12).* J Public Health (Oxf), 2016. **38**(3): p. 585-590.

33. Bauermeister, J.A., et al., *Development of a Tailored HIV Prevention Intervention for Single Young Men Who Have Sex With Men Who Meet Partners Online: Protocol for the myDEx Project.* JMIR Res Protoc, 2017. **6**(7): p. e141.

34. Billings, D.W., et al., *A Randomized Trial to Evaluate the Efficacy of a Web-Based HIV Behavioral Intervention for High-Risk African American Women.* AIDS Behav, 2015. **19**(7): p. 1263-74.

35. Brady, S.S., et al., *An Interactive Website to Reduce Sexual Risk Behavior: Process Evaluation of TeensTalkHealth.* Jmir Research Protocols, 2015. **4**(3).

36. Danielson, C.K., et al., *SiHLEWeb.com: Development and usability testing of an evidence-based HIV prevention website for female African-American adolescents.* Health Informatics Journal, 2016. **22**(2): p. 194-208.

37. Fernandez, M.I., et al., *A Randomized Controlled Trial of POWER: An Internet-Based HIV Prevention Intervention for Black Bisexual Men.* AIDS Behav, 2016. **20**(9): p. 1951-60.

38. Greene, G.J., et al., *Implementation and Evaluation of the Keep It Up! Online Hiv Prevention Intervention in a Community-Based Setting.* Aids Education and Prevention, 2016. **28**(3): p. 231-245.

39. Motley, D.N., S. Hammond, and B. Mustanski, *Strategies Chosen by YMSM During Goal Setting to Reduce Risk for HIV and Other Sexually Transmitted Infections: Results From the Keep It Up! 2.0 Prevention Trial.* AIDS Educ Prev, 2017. **29**(1): p. 1-13.

40. Mustanski, B., et al., *Internet-Based HIV Prevention With At-Home Sexually Transmitted Infection Testing for Young Men Having Sex With Men: Study Protocol of a Randomized Controlled Trial of Keep It Up! 2.0.* JMIR Res Protoc, 2017. **6**(1): p. e1.

41. Mustanski, B., et al., *Biomedical and Behavioral Outcomes of Keep It Up!: An eHealth HIV Prevention Program RCT.* Am J Prev Med, 2018. **55**(2): p. 151-158.

42. Manavi, K. and J. Hodson, *Observational study of factors associated with return of home sampling kits for sexually transmitted infections requested online in the UK.* Bmj Open, 2017. **7**(10).

43. Jones, R., L.J. Lacroix, and K. Nolte, *"Is Your Man Stepping Out?" An Online Pilot Study to Evaluate Acceptability of a Guide-Enhanced HIV Prevention Soap Opera Video Series and Feasibility of Recruitment by Facebook Advertising.* J Assoc Nurses AIDS Care, 2015. **26**(4): p. 368-86.

44. Kasatpibal, N., et al., *Effects of Internet-based instruction on HIV-prevention knowledge and practices among men who have sex with men.* Nursing & Health Sciences, 2014. **16**(4): p. 514-520.

45. Klein, C.H., et al., *C-SAFE: A Computer-Delivered Sexual Health Promotion Program for Latinas.* Health Promot Pract, 2017. **18**(4): p. 516-525.

46. Maksut, J.L., et al., *A Test of Concept Study of At-Home, Self-Administered HIV Testing With Web-Based Peer Counseling Via Video Chat for Men Who Have Sex With Men.* JMIR Public Health Surveill, 2016. **2**(2): p. e170.

47. Mustanski, B., et al., *Feasibility, Acceptability, and Initial Efficacy of an Online Sexual Health Promotion Program for LGBT Youth: The Queer Sex Ed Intervention.* Journal of Sex Research, 2015. **52**(2): p. 220-230.

48. Platteau, T., et al., *Swab2know: An HIV-Testing Strategy Using Oral Fluid Samples and Online Communication of Test Results for Men Who Have Sex With Men in Belgium.* J Med Internet Res, 2015. **17**(9): p. e213.

49. Loos, J., et al., *Acceptability of a Community-Based Outreach HIV-Testing Intervention Using Oral Fluid Collection Devices and Web-Based HIV Test Result Collection Among Sub-Saharan African Migrants: A Mixed-Method Study.* JMIR Public Health Surveill, 2016. **2**(2): p. e33.

50. Widman, L., et al., *Projectheartforgirls.Com: Development of a Web-Based Hiv/Std Prevention Program for Adolescent Girls Emphasizing Sexual Communication Skills.* Aids Education and Prevention, 2016. **28**(5): p. 365-377.

51. Wilson, E., et al., *Can Internet-Based Sexual Health Services Increase Diagnoses of Sexually Transmitted Infections (STI)? Protocol for a Randomized Evaluation of an Internet-Based STI Testing and Results Service.* JMIR Res Protoc, 2016. **5**(1): p. e9.

52. Wilson, E., et al., *Internet-accessed sexually transmitted infection (e-STI) testing and results service: A randomised, single-blind, controlled trial.* PLoS Med, 2017. **14**(12): p. e1002479.

53. Ybarra, M.L., et al., *A Randomized Controlled Trial to Increase HIV Preventive Information, Motivation, and Behavioral Skills in Ugandan Adolescents.* Ann Behav Med, 2015. **49**(3): p. 473-85.

54. Cote, J., et al., *Virtual intervention to support self-management of antiretroviral therapy among people living with HIV.* J Med Internet Res, 2015. **17**(1): p. e6.

55. Cruess, D.G., et al., *A Randomized Clinical Trial of a Brief Internet-based Group Intervention to Reduce Sexual Transmission Risk Behavior Among HIV-Positive Gay and Bisexual Men.* Ann Behav Med, 2018. **52**(2): p. 116-129.

56. Green, S.M., E. Lockhart, and S.L. Marhefka, *Advantages and disadvantages for receiving Internet-based HIV/AIDS interventions at home or at community-based organizations.* AIDS Care, 2015. **27**(10): p. 1304-8.

57. Hirshfield, S., et al., *Developing a Video-Based eHealth Intervention for HIV-Positive Gay, Bisexual, and Other Men Who Have Sex with Men: Study Protocol for a Randomized Controlled Trial.* JMIR Res Protoc, 2016. **5**(2): p. e125.

58. Horvath, K.J., et al., *Thrive With Me: Protocol for a Randomized Controlled Trial to Test a Peer Support Intervention to Improve Antiretroviral Therapy Adherence Among Men Who Have Sex With Men.* JMIR Res Protoc, 2018. **7**(5): p. e10182.

59. Mi, G., et al., *Effects of a Quasi-Randomized Web-Based Intervention on Risk Behaviors and Treatment Seeking Among HIV-Positive Men Who Have Sex With Men in Chengdu, China.* Curr HIV Res, 2015. **13**(6): p. 490-6.

60. Milam, J., et al., *Randomized Controlled Trial of an Internet Application to Reduce HIV Transmission Behavior Among HIV Infected Men Who have Sex with Men.* Aids and Behavior, 2016. **20**(6): p. 1173-1181.

61. Millard, T., et al., *Informing the development of an online self-management program for men living with HIV: a needs assessment.* Bmc Public Health, 2014. **14**.

62. Millard, T., et al., *Online self-management for gay men living with HIV: a pilot study.* Sexual Health, 2015. **12**(4): p. 308-314.

63. Miranda, J. and J. Cote, *The Use of Intervention Mapping to Develop a Tailored Web-Based Intervention, Condom-HIM.* JMIR Public Health Surveill, 2017. **3**(2): p. e20.

64. Peterson, J., et al., *Technology use in linking criminal justice reentrants to HIV care in the community: a qualitative formative research study.* J Health Commun, 2015. **20**(3): p. 245-51.

65. Anand, T., et al., *Innovative strategies using communications technologies to engage gay men and other men who have sex with men into early HIV testing and treatment in Thailand.* J Virus Erad, 2015. **1**(2): p. 111-5.

66. Anand, T., et al., *A novel Online-to-Offline (O2O) model for pre-exposure prophylaxis and HIV testing scale up.* J Int AIDS Soc, 2017. **20**(1): p. 1-11.

67. Haas, S.M., et al., *THE KNOW*NOW PROJECT: FACILITATED SEROSORTING IN HIV-STATUS SEXUAL PARTNER COMMUNICATION.* Aids Education and Prevention, 2017. **29**(5): p. 432-442.

68. Klein, C.H., et al., *Preliminary Findings of a Technology-Delivered Sexual Health Promotion Program for Black Men Who Have Sex With Men: Quasi-Experimental Outcome Study.* JMIR Public Health Surveill, 2017. **3**(4): p. e78.

69. Lau, J.T., et al., *A Randomized Control Trial for Evaluating Efficacies of Two Online Cognitive Interventions With and Without Fear-Appeal Imagery Approaches in Preventing Unprotected Anal Sex Among Chinese Men Who Have Sex with Men.* AIDS Behav, 2016. **20**(9): p. 1851-62.

70. Marsch, L.A., et al., *Comparative Effectiveness of Web-Based vs. Educator-Delivered HIV Prevention for Adolescent Substance Users: A Randomized, Controlled Trial.* J Subst Abuse Treat, 2015. **59**: p. 30-7.

71. Mitchell, J.W., et al., *HIV-discordant and concordant HIV-positive male couples' recommendations for how an eHealth HIV prevention toolkit for concordant HIV-negative male couples could be improved to meet their specific needs.* AIDS Care, 2018. **30**(sup2): p. 54-60.

72. Stephenson, R., et al., *Home-Based HIV Testing and Counseling for Male Couples (Project Nexus): A Protocol for a Randomized Controlled Trial.* JMIR Res Protoc, 2017. **6**(5): p. e101.

73. van den Berg, J.J., et al., *Using eHealth to Reach Black and Hispanic Men Who Have Sex With Men Regarding Treatment as Prevention and Preexposure Prophylaxis: Protocol for a Small Randomized Controlled Trial.* JMIR Res Protoc, 2018. **7**(7): p. e11047.

74. De Boni, R.B., et al., *Self-testing, communication and information technology to promote HIV diagnosis among young gay and other men who have sex with men (MSM) in Brazil.* J Int AIDS Soc, 2018. **21 Suppl 5**: p. e25116.

75. Dolwick Grieb, S.M., et al., *'Vive': Designing an Intervention to Improve Timely HIV Diagnosis Among Latino Immigrant Men.* Progress in Community Health Partnerships-Research Education and Action, 2016. **10**(3): p. 365-372.

76. Dolwick Grieb, S.M., A. Flores-Miller, and K.R. Page, *¡Solo Se Vive Una Vez! (You Only Live Once): A Pilot Evaluation of Individually Tailored Video Modules Aiming to Increase HIV Testing Among Foreign-Born Latino Men.* J Acquir Immune Defic Syndr, 2017. **74 Suppl 2**: p. S104-S112.

77. Stephenson, R., et al., *Providing Home-Based HIV Testing and Counseling for Transgender Youth (Project Moxie): Protocol for a Pilot Randomized Controlled Trial.* JMIR Res Protoc, 2017. **6**(11): p. e237.

78. Cho, H., et al., *Understanding the predisposing, enabling, and reinforcing factors influencing the use of a mobile-based HIV management app: A real-world usability evaluation.* Int J Med Inform, 2018. **117**: p. 88-95.

79. Cho, H., et al., *A Multi-Level Usability Evaluation of Mobile Health Applications: A Case Study.* J Biomed Inform, 2018.

80. Schnall, R., et al., *Mobile Health Technology for Improving Symptom Management in Low Income Persons Living with HIV.* AIDS Behav, 2018.

81. Chiu, C.J., et al., *Ethics issues in social media-based HIV prevention in low- and middle-income countries.* Camb Q Healthc Ethics, 2015. **24**(3): p. 303-10.

82. Garett, R., L. Menacho, and S.D. Young, *Ethical Issues in Using Social Media to Deliver an HIV Prevention Intervention: Results from the HOPE Peru Study.* Prev Sci, 2017. **18**(2): p. 225-232.

83. Young, S.D., *Social media technologies for HIV prevention study retention among minority men who have sex with men (MSM).* AIDS Behav, 2014. **18**(9): p. 1625-9.

84. Young, S.D., et al., *Social networking technologies as an emerging tool for HIV prevention: a cluster randomized trial.* Ann Intern Med, 2013. **159**(5): p. 318-24.

85. Young, S.D., et al., *The HOPE social media intervention for global HIV prevention in Peru: a cluster randomised controlled trial.* Lancet HIV, 2015. **2**(1): p. e27-32.

86. Young, S.D., et al., *Project HOPE: online social network changes in an HIV prevention randomized controlled trial for African American and Latino men who have sex with men.* Am J Public Health, 2014. **104**(9): p. 1707-12.

87. Alarcon Gutierrez, M., et al., *Acceptability and effectiveness of using mobile applications to promote HIV and other STI testing among men who have sex with men in Barcelona, Spain.* Sex Transm Infect, 2018.

88. Bauermeister, J., et al., *Reducing HIV Vulnerability Through a Multilevel Life Skills Intervention for Adolescent Men (The iREACH Project): Protocol for a Randomized Controlled Trial.* JMIR Res Protoc, 2018. **7**(7): p. e10174.

89. Huang, E., et al., *Using Grindr, a Smartphone Social-Networking Application, to Increase HIV Self-Testing Among Black and Latino Men Who Have Sex With Men in Los Angeles, 2014.* AIDS Educ Prev, 2016. **28**(4): p. 341-50.

90. Jenkins Hall, W., et al., *HIV-Prevention Opportunities With GPS-Based Social and Sexual Networking Applications for Men Who Have Sex With Men.* AIDS Educ Prev, 2017. **29**(1): p. 38-48.

91. Lampkin, D., et al., *Reaching Suburban Men Who Have Sex With Men for STD and HIV Services Through Online Social Networking Outreach: A Public Health Approach.* J Acquir Immune Defic Syndr, 2016. **72**(1): p. 73-8.

92. Lelutiu-Weinberger, C., et al., *Feasibility, Acceptability, and Preliminary Efficacy of a Live-Chat Social Media Intervention to Reduce HIV Risk Among Young Men Who Have Sex With Men.* AIDS Behav, 2015. **19**(7): p. 1214-27.

93. Patel, V.V., et al., *Empowering With PrEP (E-PrEP), a Peer-Led Social Media-Based Intervention to Facilitate HIV Preexposure Prophylaxis Adoption Among Young Black and Latinx Gay and Bisexual Men: Protocol for a Cluster Randomized Controlled Trial.* JMIR Res Protoc, 2018. **7**(8): p. e11375.

94. Rhodes, S.D., et al., *Using Social Media to Increase HIV Testing Among Gay and Bisexual Men, Other Men Who Have Sex With Men, and Transgender Persons: Outcomes From a Randomized Community Trial.* Clinical Infectious Diseases, 2016. **62**(11): p. 1450-1453.

95. Sun, C.J., et al., *Acceptability and feasibility of using established geosocial and sexual networking mobile applications to promote HIV and STD testing among men who have sex with men.* AIDS Behav, 2015. **19**(3): p. 543-52.

96. Sun, W.H., C.K.H. Wong, and W.C.W. Wong, *A Peer-Led, Social Media-Delivered, Safer Sex Intervention for Chinese College Students: Randomized Controlled Trial.* Journal of Medical Internet Research, 2017. **19**(8).

97. Tucker, J.D., et al., *Crowdsourcing to promote HIV testing among MSM in China: study protocol for a stepped wedge randomized controlled trial.* Trials, 2017. **18**.

98. Tang, W., et al., *Crowdsourcing to expand HIV testing among men who have sex with men in China: A closed cohort stepped wedge cluster randomized controlled trial.* PLoS Med, 2018. **15**(8): p. e1002645.

99. Washington, T.A., S. Applewhite, and W. Glenn, *Using Facebook as a Platform to Direct Young Black Men Who Have Sex With Men to a Video-Based HIV Testing Intervention: A Feasibility Study.* Urban Soc Work, 2017. **1**(1): p. 36-52.

100. Tanner, A.E., et al., *Wecare: A Social Media-Based Intervention Designed to Increase Hiv Care Linkage, Retention, and Health Outcomes for Racially and Ethnically Diverse Young Msm.* Aids Education and Prevention, 2016. **28**(3): p. 216-230.

101. Baltierra, N.B., et al., *More than just tracking time: Complex measures of user engagement with an internet-based health promotion intervention.* J Biomed Inform, 2016. **59**: p. 299-307.

102. Bauermeister, J.A., et al., *HIV and Sexuality Stigma Reduction Through Engagement in Online Forums: Results from the HealthMPowerment Intervention.* AIDS Behav, 2018.

103. Hightow-Weidman, L., et al., *healthMpowerment: effects of a mobile phone-optimized, Internet-based intervention on condomless anal intercourse among young black men who have sex with men and transgender women*, in *9th IAS Conference on HIV Science (IAS 2017)*. 2017: Paris, France.

104. Hightow-Weidman, L.B., et al., *HealthMpowerment.org: Building Community Through a Mobile-Optimized, Online Health Promotion Intervention.* Health Educ Behav, 2015. **42**(4): p. 493-9.

105. Hightow-Weidman, L., et al., *A Gamified Smartphone App to Support Engagement in Care and Medication Adherence for HIV-Positive Young Men Who Have Sex With Men (AllyQuest): Development and Pilot Study.* JMIR Public Health Surveill, 2018. **4**(2): p. e34.

106. LeGrand, S., et al., *Epic Allies: Development of a Gaming App to Improve Antiretroviral Therapy Adherence Among Young HIV-Positive Men Who Have Sex With Men.* JMIR Serious Games, 2016. **4**(1): p. e6.

107. LeGrand, S., et al., *Epic Allies, a Gamified Mobile Phone App to Improve Engagement in Care, Antiretroviral Uptake, and Adherence Among Young Men Who Have Sex With Men and Young Transgender Women Who Have Sex With Men: Protocol for a Randomized Controlled Trial.* JMIR Res Protoc, 2018. **7**(4): p. e94.

108. Whiteley, L., et al., *A Mobile Gaming Intervention to Increase Adherence to Antiretroviral Treatment for Youth Living With HIV: Development Guided by the Information, Motivation, and Behavioral Skills Model.* JMIR Mhealth Uhealth, 2018. **6**(4): p. e96.

109. Shegog, R., et al., *NATIVE-It's Your Game: Adapting a Technology-Based Sexual Health Curriculum for American Indian and Alaska Native youth.* J Prim Prev, 2017. **38**(1-2): p. 27-48.

110. Schonnesson, L.N., A.M. Bowen, and M.L. Williams, *Project SMART: Preliminary Results From a Test of the Efficacy of a Swedish Internet-Based HIV Risk-Reduction Intervention for Men Who Have Sex With Men.* Arch Sex Behav, 2016. **45**(6): p. 1501-11.

111. Fiellin, L.E., et al., *Video Game Intervention for Sexual Risk Reduction in Minority Adolescents: Randomized Controlled Trial.* J Med Internet Res, 2017. **19**(9): p. e314.

112. Montanaro, E., et al., *Using Videogame Apps to Assess Gains in Adolescents' Substance Use Knowledge: New Opportunities for Evaluating Intervention Exposure and Content Mastery.* J Med Internet Res, 2015. **17**(10): p. e245.

113. Fiellin, L.E., et al., *The design and implementation of a randomized controlled trial of a risk reduction and human immunodeficiency virus prevention videogame intervention in minority adolescents: PlayForward: Elm City Stories.* Clin Trials, 2016. **13**(4): p. 400-8.

114. Lukhele, B.W., et al., *Efficacy of Mobile Serious Games in Increasing HIV Risk Perception in Swaziland: A Randomized Control Trial (SGprev Trial) Research Protocol.* JMIR Res Protoc, 2016. **5**(4): p. e224.

115. Enah, C., K. Piper, and L. Moneyham, *Qualitative Evaluation of the Relevance and Acceptability of a Web-Based HIV Prevention Game for Rural Adolescents.* Journal of Pediatric Nursing-Nursing Care of Children & Families, 2015. **30**(2): p. 321-328.
