# Supplementary figures and images for: Electronic and other new media technology interventions for HIV care and prevention: a systematic review
Source: J Int AIDS Soc. 2020 Jan 7;23(1):e25439. doi: 10.1002/jia2.25439 (PMC6945883; doi:10.1002/jia2.25439)

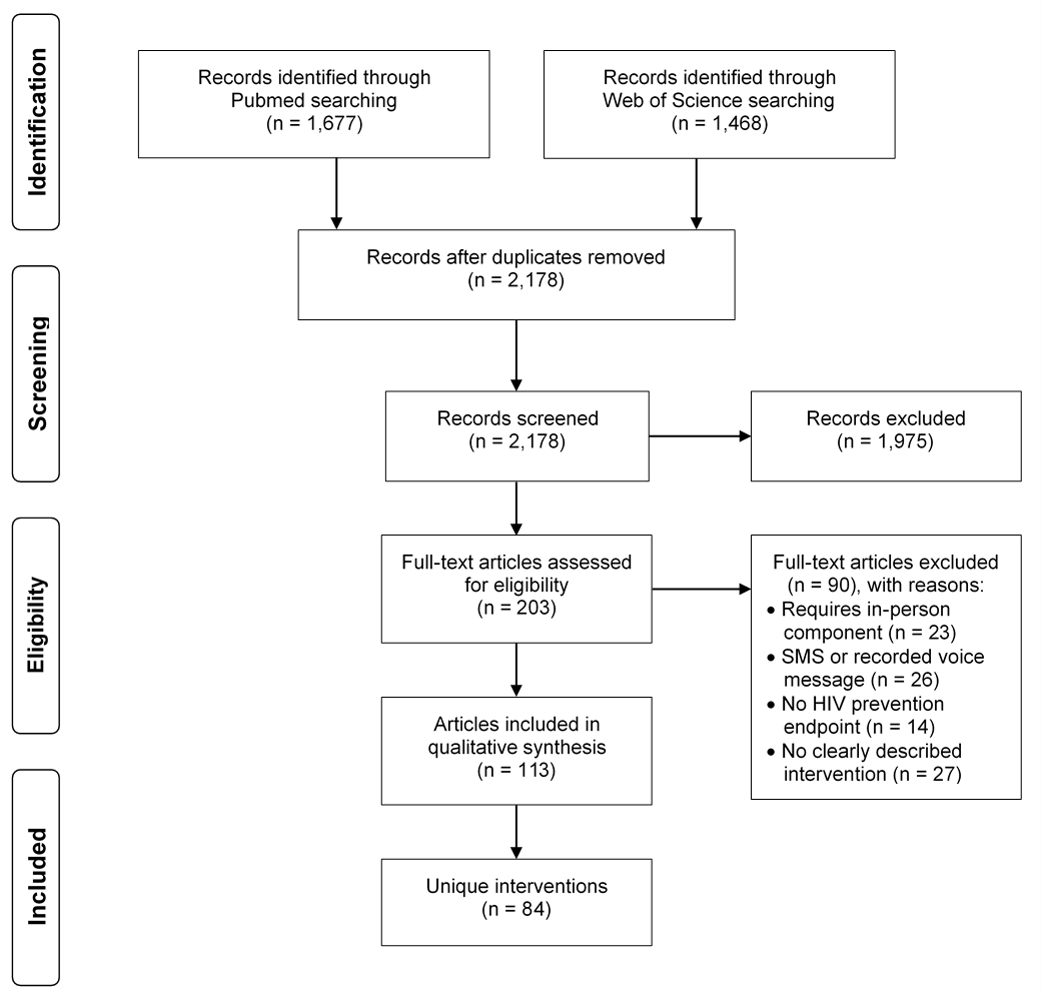

Supplement: Supplementary file 2 — Figure S1. Results of the database search for recently published eHealth interventions. [file JIA2-23-e25439-s001.png]
